# Application of 5-Fluorouracil-Polycaprolactone Sustained-Release Film in Ahmed Glaucoma Valve Implantation Inhibits Postoperative Bleb Scarring in Rabbit Eyes

**DOI:** 10.1371/journal.pone.0141467

**Published:** 2015-11-18

**Authors:** Xiu-Zeng Bi, Wei-Hua Pan, Xin-Ping Yu, Zong-Ming Song, Zeng-Jin Ren, Min Sun, Cong-Hui Li, Kai-Hui Nan

**Affiliations:** 1 Hospital of Optometry and Ophthalmology, Wenzhou Medical College, Wenzhou, 325027, Zhejiang Province, China; 2 School of Optometry and Ophthalmology, Wenzhou Medical College, Wenzhou, 325027, Zhejiang Province, China; 3 Wenzhou Engineering Research Center for Biomaterials, Wenzhou, 325027, Zhejiang Province, China; Tohoku University, JAPAN

## Abstract

This study was designed to investigate whether 5-fluorouracil (5-Fu)-polycaprolactone sustained-release film in Ahmed glaucoma valve implantation inhibits postoperative bleb scarring in rabbit eyes. Eighteen New Zealand white rabbits were randomly divided into three groups (A, B and C; n = 6 per group). Group A received combined 5-Fu-polycaprolactone sustained-release film application and Ahmed glaucoma valve implantation, group B received local infiltration of 5-Fu and Ahmed glaucoma valve implantation, and group C received Ahmed glaucoma valve implantation. Postoperative observations were made of the anterior segment, intraocular pressure, central anterior chamber depth, blebs, drainage tube, and accompanying ciliary body detachment. The pathology of the blebs and surrounding tissues were observed at month 3 postoperatively. We revealed that the 5-Fu-polycaprolactone sustained-release film maintained a release concentration range of 13.7 ± 0.12 to 37.41 ± 0.47 μg/ml over three months *in vitro*. Postoperatively, diffuse blebs with ridges were found in all eyes in group A, two blebs were observed in group B, and no bleb formation was present in group C. The postoperative central anterior chamber depth in group A was significantly less than that of the other two groups. The postoperative intraocular pressure of group A stabilized at 6.33–8.67 mmHg, whereas that of group C gradually remained at 7.55–10.02 mmHg. The histopathology showed that the fibrous tissue thickness of the blebs in group A was significantly thinner than that of the other groups. We conclude that the 5-Fu-polycaprolactone sustained-release film had a sustained drug release effect, which promoted the inhibition of bleb scarring after Ahmed glaucoma valve implantation.

## Introduction

Ahmed glaucoma valve (AGV), which was granted clearance for sale by the Food and Drug Administration (FDA) in 1993, has become the preferred treatment for refractory glaucoma. Clinical studies have confirmed that compared with other aqueous humor drainage devices, the AGV (FP7/8) has a relatively lower incidence of postoperative complications [1,2]. The success rate of AGV implantation is approximately 80% one year after the operation, but the five-year success rate drops to 40–50%, of which approximately 30% of patients suffer postoperative bleb scarring [3]. Among the various medications administered to prevent scarring, 5-fluorouracil (5-Fu) was confirmed to inhibit bleb scarring in trabeculectomy [4]. However, a single application of 5-Fu cannot effectively reduce postoperative bleb fibrosis in AGV implantation [5]. The use of multiple local injections of 5-Fu to reduce bleb fibrosis increases the risks of corneal damage, bleb leakage, continuous low intraocular pressure (IOP), intraocular infection, and other complications [6]. As a result, it is essential to determine a safe and effective drug release tool to improve the success rate of AGV implantation.

Polycaprolactone [(Poly (ε-Caprolactone), PCL] is a novel biodegradable polymer material, which has been prepared as sustained-release microspheres[7], intraocular implants[8], and sustained-release tablets [9]. No apparent side effects of PCL were found in animal studies [8]. However, these reports described several disadvantages of using PCL as a drug release tool, including its complicated preparation procedure, low drug concentration, and clear drug burst release phenomenon [7]. In addition, few histopathological studies have evaluated the inhibition of scarring after AGV implantation.

Taking advantage of PCL’s film-forming property, we prepared a 5-Fu-PCL sustained-release film using spray-forming methods, and observed the release effects of the film *in vitro*. We then investigated the *in vivo* inhibitory effects of 5-Fu-PCL sustained-release film on bleb scarring after AGV implantation in rabbit eyes.

## Material and Methods

All animals were treated according to the ARVO Statement for the Use of Animals in Ophthalmic and Vision Research, and protocols were approved by the Institutional Animal Care and Use Committee in Wenzhou Medical University. The animals were housed in an air-conditioned room with an ambient temperature of 16–26°C, a relative humidity of 40–70% and a 12-hour light-dark cycle with a daytime light intensity of approximately 500 lux. Rabbits were provided with a commercial primate diet. In addition, fresh vegetables were provided twice daily, and water was freely available at all times. All examinations were performed gently and all efforts were made to minimize suffering. Topical proparacaine HCl (0.5% Alcaine, Alcon Laboratories, Ft. Worth, TX) was used before measuring IOP, AS-OCT and slit-lamp examination. All surgical manipulations were carried out under general anesthesia induced with an intramuscular injection of 0.25 ml/kg Sumianxin II (China Animal Husbandry Animal Health Ltd., Changchun, China) and 1 ml/kg of 3% sodium pentobarbital (Beijing Chemical Reagent Company, Beijing, China). The health of these animals was monitored every day after surgery through observing and analyzing the growth vigor, diet and activity. Tobradex ointment (Dexamethasone/tobramycin Eye Ointment, Alcon Laboratories, Ft. Worth, TX) was used three times for 7 days to alleviate suffering during post-surgery recovery. Animals were sacrificed with an intravenous injection of an overdose of sodium pentobarbital solution (Beijing Chemical Reagent Company, Beijing, China).

### Preparation of 5-Fu-PCL sustained-release film

5-Fu (Aladdin, Shanghai, China) was ultrasonically dispersed in 5 ml of acetonitrile, and PCL (Sigma-Aldrich, California, USA) was ultrasonically dissolved in 15 ml of dichloromethane. After mixing, the solution was sprayed using a LPH-50 spray gun (ANEST IWATA, Japan) on a polytetrafluoroethylene plate with nitrogen stream at 0.2 MPa. After vacuum drying for 12 h, the resultant sustained-release film was cut using a mold to a diameter of 6 mm and a thickness of 0.35 ± 0.01 mm (mean ± standard deviation [SD]). The drug loading of the prepared 5-Fu-PCL sustained-release film was 4.13 ± 0.12 mg.

### In vitro release detection of 5-Fu-PCL sustained-release film

As previously reported, three parallel samples of the 5-Fu-PCL sustained-release film were respectively placed in a dialysis bag (MD 34 mm, 7000) and put into a centrifuge tube filled with 10 ml phosphate buffered saline (PBS; pH = 7.4) [10]. Standard 5-Fu (National Institutes for Food and Drug Control, China) was used as the control. The tubes were then placed in a shaker (37°C, 100 revolutions/minute). Samples of 4 ml were taken at different time points (1, 2, 4, 8 and12h, 1, 2, 3, 4, 5, 6, 7, 10, 14, 21, 28, 35, 42, 49, 56, 63, 70, 77, 84 and 91d) and replaced with an equal amount of fresh PBS [10]. The *in vitro* drug release of the sustained-release film was determined by measuring the concentrations of 5-Fu in the different samples using high-performance liquid chromatography (HPLC) [11]. Each measurement was repeated three times.

### Animals

Eighteen healthy New Zealand white rabbits weighing 2.0–2.5 kg were randomly divided into three groups (A, B and C, n = 6 per group) using a random number table. In each animal, an operation was performed on the right eye. Group A received combined 5-Fu-PCL sustained-release film application and AGV implantation. Group B received local infiltration of 5-Fu and AGV implantation. Group C received AGV implantation. All animals were given adaptive feeding for 3 days. Binocular IOP was measured with a Tono-Pen tonometer (TPA, Reichert Inc., NY, USA). The depths of the anterior segment and the central anterior chamber, as well as the degree of ciliary body detachment were evaluated by anterior segment optical coherence tomography (AS-OCT, Carl Zeiss, USA) [12].

### Surgical procedures

**Fig 1:**
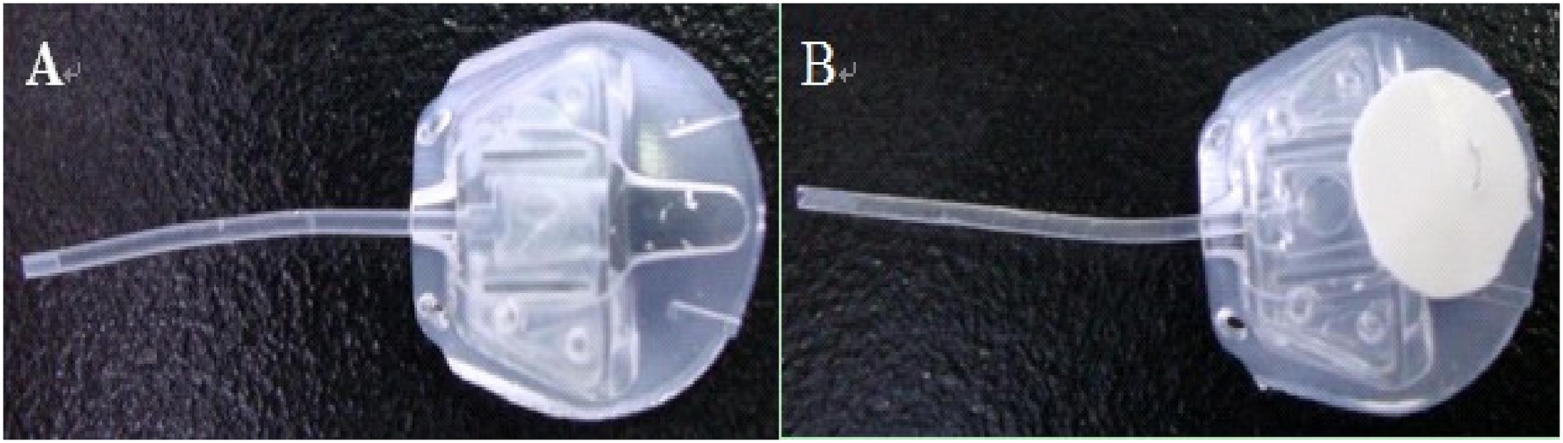
(A) AGV (New World Medical, Inc., USA, FP8). (B) The 5-Fu-PCL sustained-release film was fixed on the AGV with a 10–0 suture.

All experimental procedures were performed by the same surgeon (Weihua Pan). After sterilization with ethylene oxide, the 5-Fu-PCL sustained-release film was fixed with a 10–0 suture on the AGV (Fig 1). The animals were anesthetized with an intramuscular injection of 0.25 ml/kg Sumianxin II (China Animal Husbandry Animal Health Ltd., Changchun, China) and 1 ml/kg of 3% sodium pentobarbital (Beijing Chemical Reagent Company, Beijing, China). After routine disinfection, the superior rectus was fixed using 4–0 Mousse line (Johnson Medical Equipment Company, USA). At 9:30–12:00, a conjunctival flap was made in the fornix, and the conjunctival and subconjunctival tissues were separated bluntly to expose the sclera equator.

In group A, AGV with the 5-Fu-PCL sustained-release film was implanted in the scleral surface. In group B, after fixing an AGV on the scleral surface, a 5 mm × 3 mm piece of cotton soaked with 25 mg/ml 5-Fu (volume 140 μl) was placed on the surface for 5 minutes, and then the surgical field was rinsed with 100 ml saline. In group C, the eyes were simply implanted with AGV. In all experimental animals, the AGV was placed between the rectus and lateral rectus, 7 mm from the front edge of the limbus. It was sutured with 2 needles using 5–0 polyester line (Alcon Laboratories, Inc. USA) and then fixed on the scleral surface. A tobramycin-dexamethasone combination (Tobradex^®^) eyedrop was given the operated eyes for one week after the operation.

### Postoperative clinical observation and histopathological examination

Postoperative observations were conducted on days 1, 3, 7 and 14, and at months 1, 2, and 3. The main measurements included IOP, anterior segment evaluation with a slit-lamp including the central anterior chamber depth, blebs, the drainage tube and accompanied ciliary body detachment as well as AS-OCT [13–15], and the posterior segment evolution by a 90 diopter (D) present lens. At 3 months postoperatively, the animals were killed by an intravenous injection of sodium pentobarbital solution. The operative eye was removed carefully so as not to damage the blebs and implants. After the vitreous cavity was filled with 10% formaldehyde fixative for 24 h, the eyeball was dissected and made into paraffin sections with a thickness of 5 μm. Hematoxylin-eosin staining and trichromatic staining were used to examine the fibrous tissue [3]. The histological biopsy was performed by a pathologist in a blinded trial. The pathology of the blebs and surrounding tissues was observed, including the baseline side (scleral surface), the roof side (conjunctival surface), the optic nerve side (the pole), and the limbus side (the front end of the bleb). The fibrous tissue thickness in the four directions was measured with a micrometer, and the differences in bleb scarring were compared among the groups.

### Statistical analysis

A general linear model was used to analyze the correlations in repeated measurements and the interactions of timing and grouping. Analysis of variance (ANOVA) was performed to compare groups at each time point. The statistical analysis was performed using SPSS16.0 software. A *P* value less than 0.05 was considered statistically significant.

## Results

**Fig 2:**
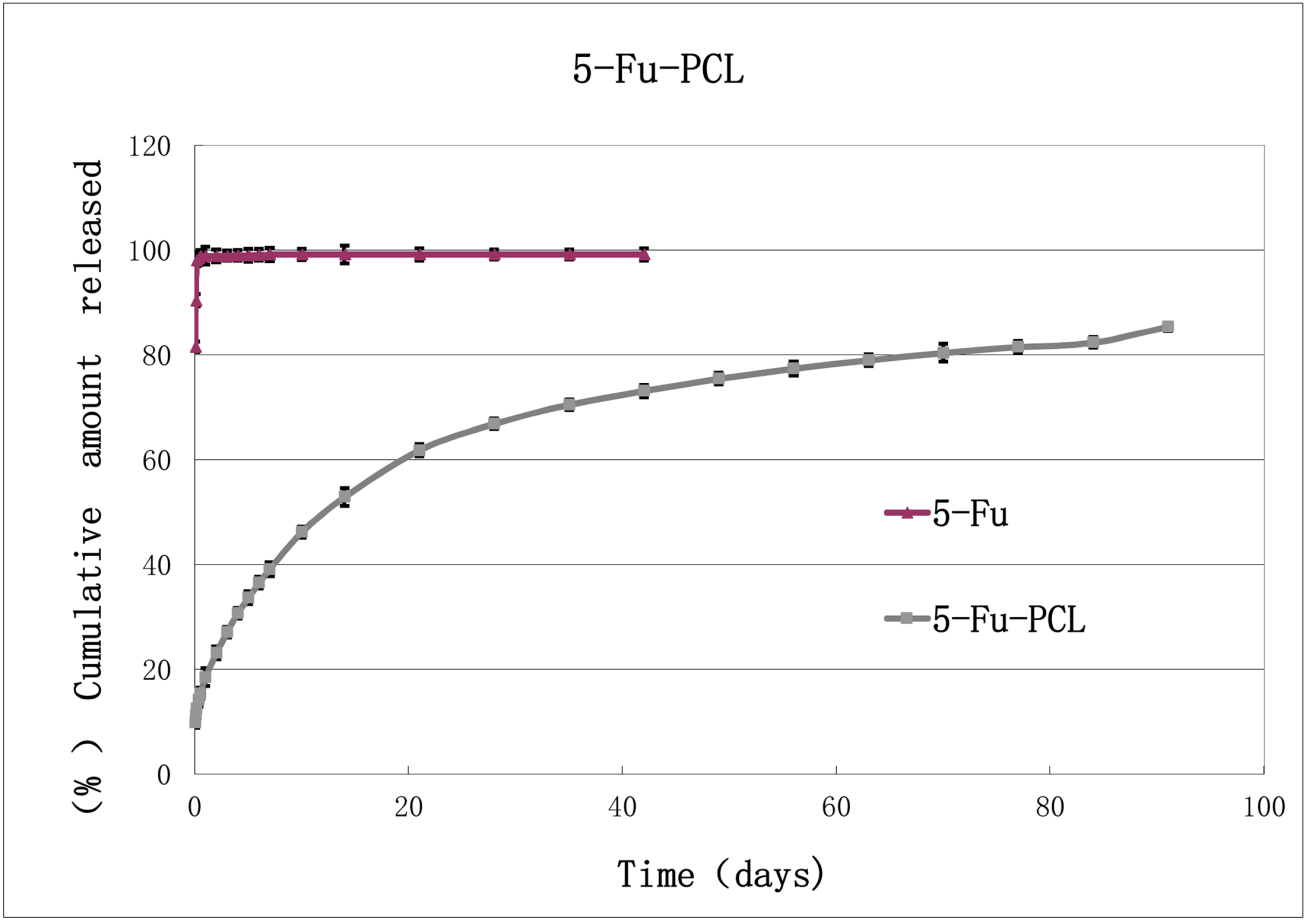
Drug-release profile of the 5-Fu-PCL film (n = 3, mean ± SD).

The *in vitro* drug release of the 5-Fu-PCL sustained-release film was maintained for at least 3 months. The regression analysis of the release pattern showed that the dissolution curve of the drug release was in accordance with the Higuchi equation: Y = 5.6758t^1/2^+9.6786, R^2^ = 0.9948. This result indicated that the 5-Fu-PCL sustained-release film had the features of a long-acting preparation. In addition, the *in vitro* drug release of the 5-Fu-PCL sustained-release film was relatively stable with a concentration range of 13.7 ± 0.12 μg/ml to 37.41 ± 0.47 μg/ml over 3 months as shown in Fig 2.

**Fig 3:**
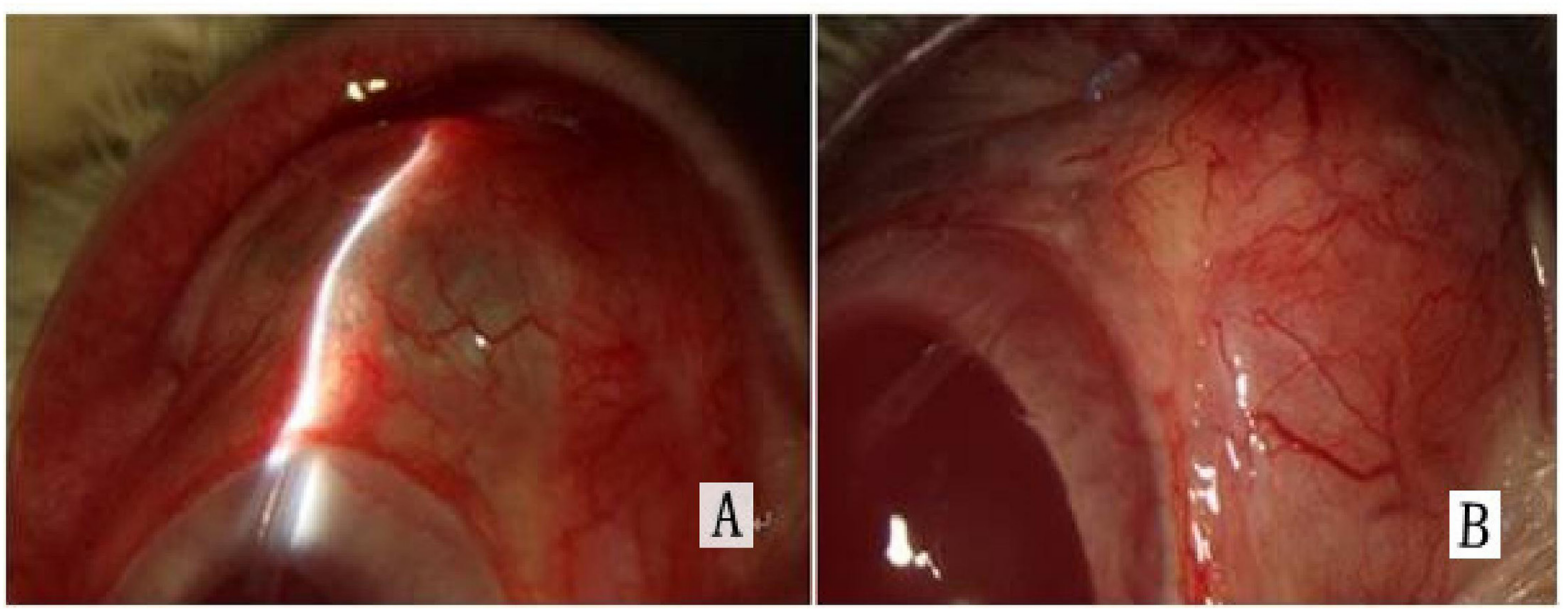
Slit-lamp microscope observations. (A) Diffuse filtering bleb in group A. (B) Flattened bleb on the AS-OCT image in groups B and C.

All animals survived from the surgical procedures. Postoperatively, no abnormalities of anterior and posterior segments were found in all animals. The location of the drainage tube in the anterior chamber was also normal, and the drainage was effective without leakage on the blebs in each animal. The blebs in group A were diffuse and had ridges (Fig 3).

**Fig 4:**
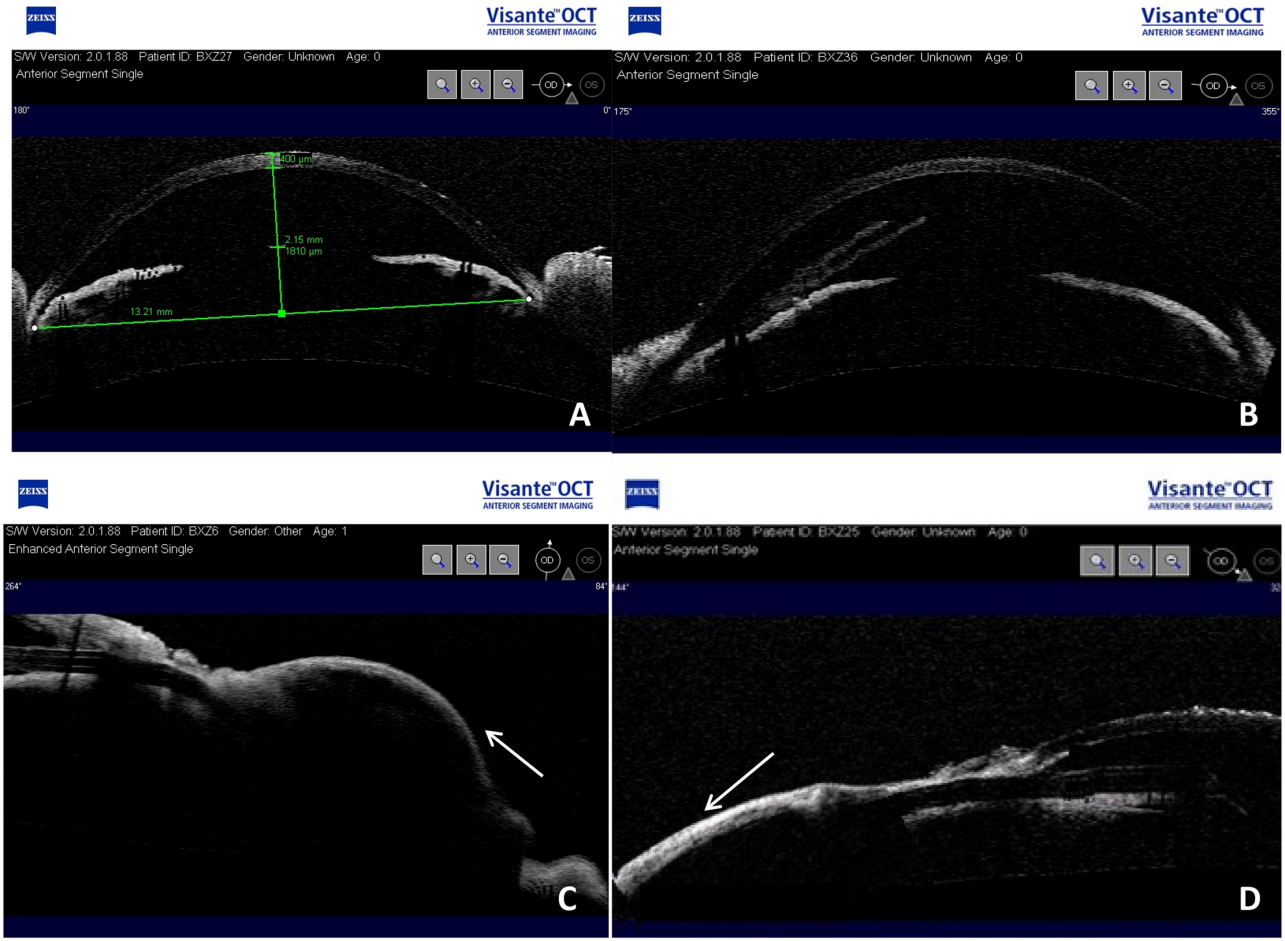
**(A) AS-OCT reveals the central depth of the anterior chamber**. (B) Drainage tube in the anterior chamber without occlusion. (C) Diffuse filtering bleb in group A. (D) Flattened bleb on the AS-OCT image in groups B and C.

As shown in Fig 4, the AS-OCT examination indicated that the AGV was located in a normal position and was unobstructed. In the third month postoperatively, diffuse blebs with ridges were found in all eyes in group A, there were two blebs in group B, and there was no bleb formation in group C. No ciliary body detachment in any direction was found in any of the groups.

**Fig 5:**
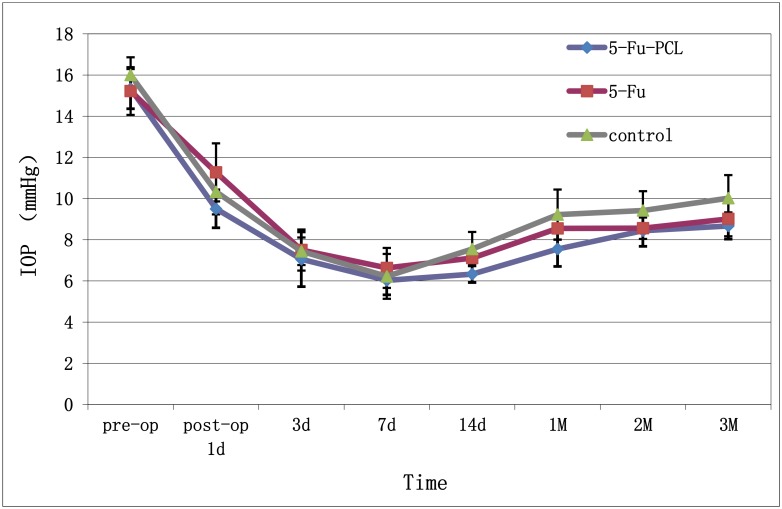
IOP after AGV implantation (mean ± SD, n = 6).

The preoperative IOP was 15.33 ± 0.97 mmHg in group A, 15.22 ± 1.16 mmHg in group B, and 16.00 ± 0.86 mmHg in group C. There were no significant differences among the three groups. By the 14th postoperative day, the IOP of group A had stabilized at 6.33–8.67 mmHg, whereas the IOP of group C had gradually increased to 7.55–10.02 mmHg. The difference in the IOP between groups A and C was statistically significant; however, the IOP in group B was not statistically different from those of the other groups (Fig 5).

**Fig 6:**
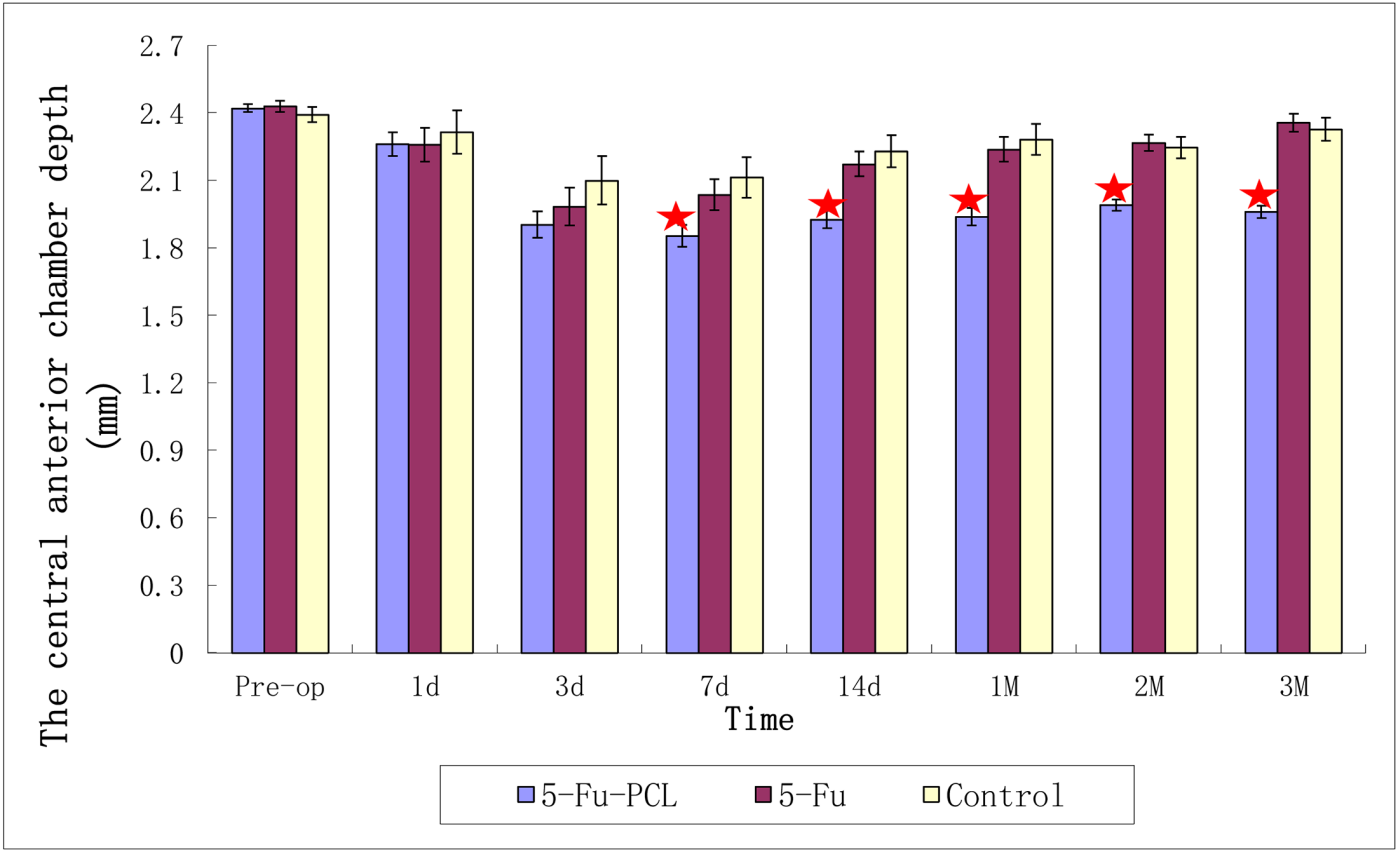
Central depth of the anterior chamber after AGV implantation. “★” *P* < 0.05 between group A and groups B and C.

The preoperative central anterior chamber depth was 2.421 ± 0.018 mm in group A, 2.429 ± 0.026 mm in group B, and 2.392 ± 0.033 mm in group C. On the 3rd month postoperatively, the central anterior chamber depth was 1.960 ± 0.028 mm in group A, which was significantly different from that of group B (2.357 ± 0.040 mm) and group C (2.327 ± 0.051 mm) (Fig 6).

**Fig 7:**
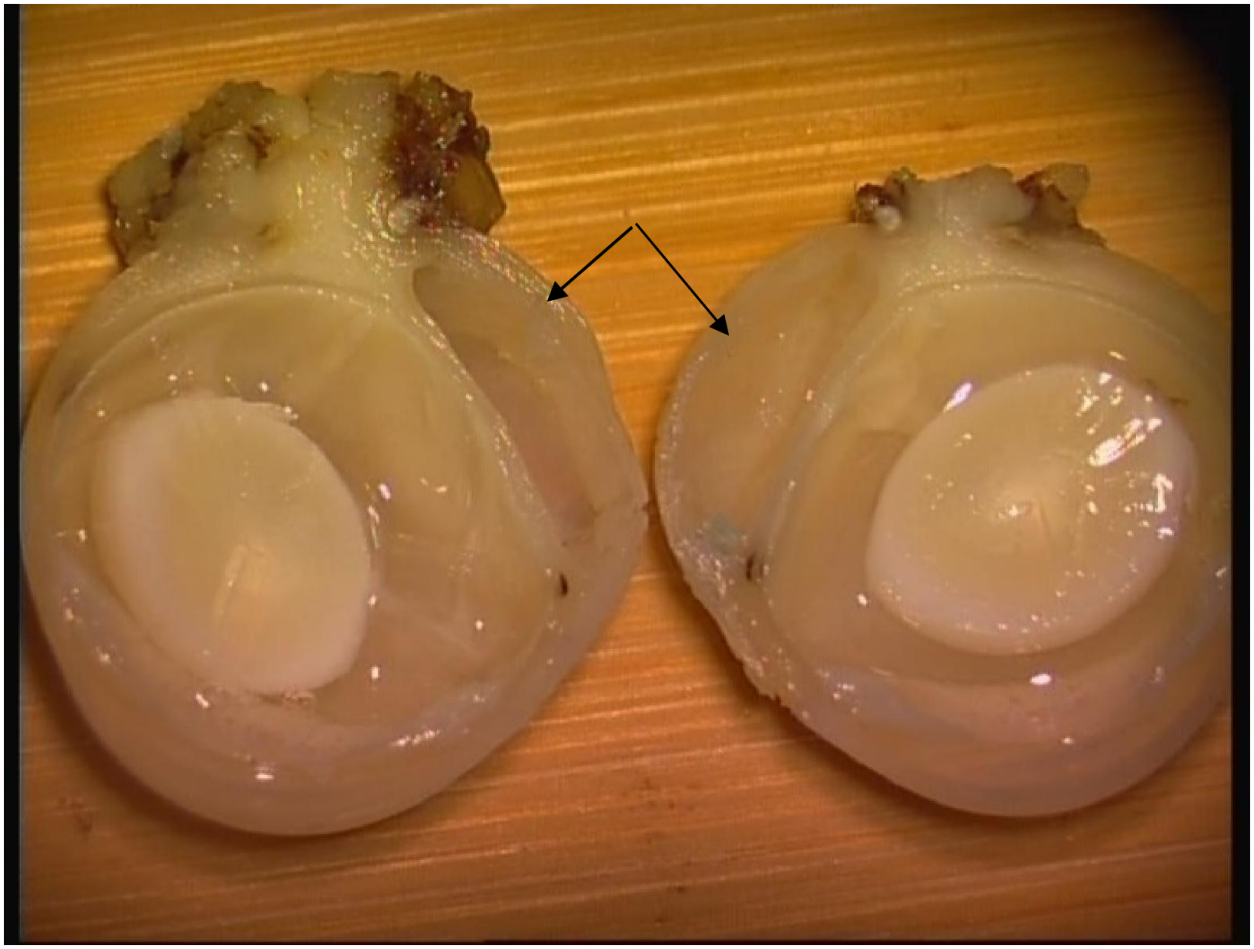
Bleb in a rabbit eye after implantation of AGV and 5-Fu-PCL sustained-release film, as indicated by the arrows.

**Fig 8:**
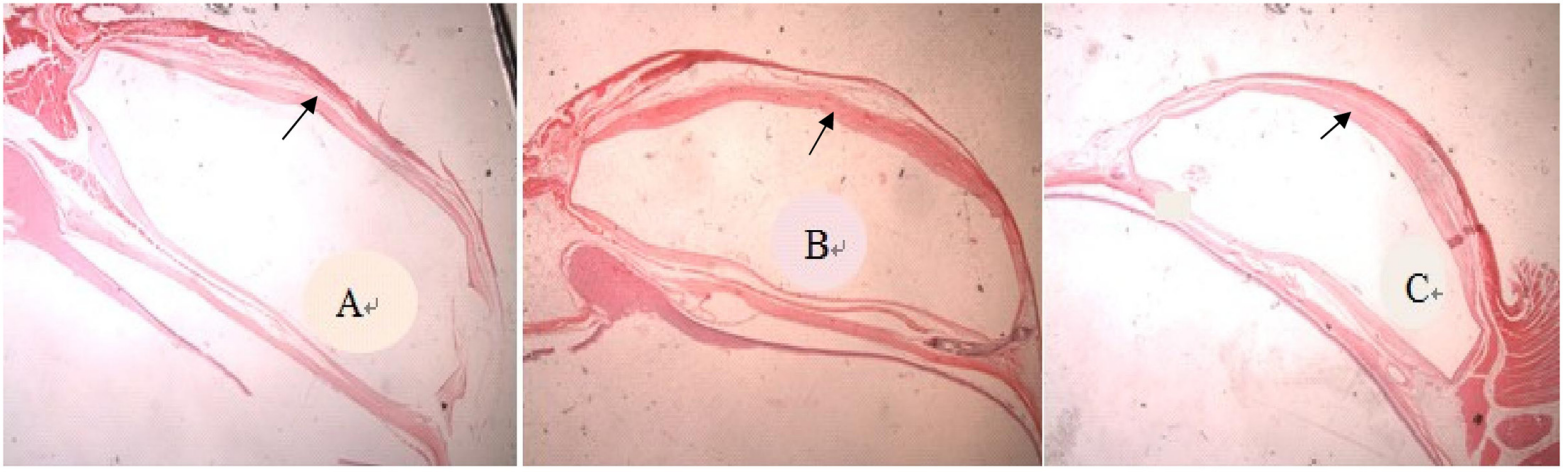
Hematoxylin-eosin staining of a bleb after AGV implantation. (A) Group A. (B) Group B. (C) Group C. Original magnification ×10.

**Fig 9:**
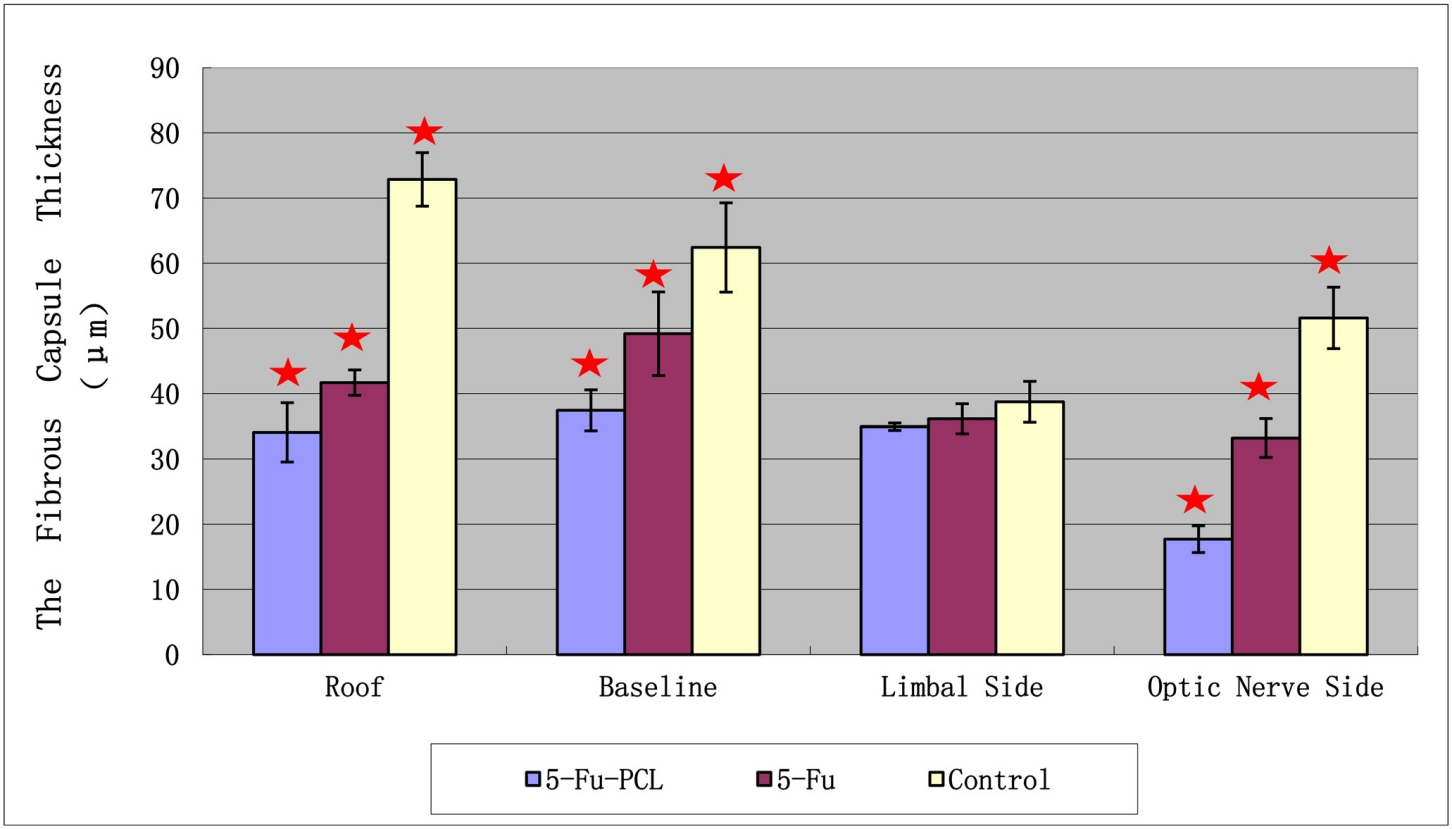
Effect of the 5-Fu-PCL sustained-release film on fibrous capsule thickness in various regions of the bleb wall. Values are presented as the mean ± standard error of the mean (SEM). “★” *P* < 0.05 between group A and groups B and C.

Fig 7 shows a bleb in one of the rabbits after implantation of AGV and 5-Fu-PCL film. The histopathology results showed that the fibrous tissue thickness of the blebs in group A was significantly thinner than that of the other groups (Fig 8). The thickness of the blebs in four directions is shown in Fig 9. The range in thickness of the blebs in the three groups was as follows: the base side was 37.44–62.43 μm, the top side was 34.08–72.87 μm, and the optical nerve side was 17.72–51.61 μm. Significant differences in bleb thickness were found among the three groups for these three sides. However, in the limbus position, the bleb thickness was 34.94 μm in group A, 36.15 μm in group B, and 38.73 μm in group C, and there were no significant differences among the groups.

## Discussion

In this study, we successfully prepared a 5-Fu-PCL sustained-release film using a spray-forming method. Along with having a simple preparation process, the 5-Fu-PCL sustained-release film has several other advantages, including sufficient drug volume, stable release, and long-duration drug delivery. Our animal experiments and pathological findings indicate that combined AGV implantation and 5-Fu-PCL sustained-release film application in rabbit eyes was safe, and this approach effectively inhibited bleb scarring after the AGV implantation.

IOP depends on the encapsulation of the drain surrounding the rear disc, the resistance of the encapsulation wall, and the surface area of the encapsulation. If the encapsulation wall is thinner or if the surface area of the encapsulation is greater, then the IOP is lower[16]. Lloyd et al.[17] showed that failed IOP control at the intermediate and late stages after AGV implantation was related to decreased bleb penetration and increased aqueous humor drainage obstruction due to excessive fibrosis surrounding the rear disc and bleb.

The *in vitro* studies performed by Leonard et al. [18] demonstrated that the thin PCL film had good biocompatibility and the ability to support the proliferation of conjunctival epithelial cells *in vitro*. These results indicated that PCL film might have potential as a human conjunctival tissue engineering scaffold material. Another report by Armando et al. [19] confirmed that a PCL carrier with a dexamethasone release agent could be successfully implanted into rabbit eyes and slowly released dexamethasone for more than 55 weeks. These results indicated the feasibility of using PCL as an intraocular, sustained-release drug carrier.

In comparison to local infiltration of liquid 5-Fu, a significant advantage of combined AGV and 5-Fu-PCL sustained-release film implantation is that it maintains functional blebs for at least three months. As confirmed by Cantor et al.[5], local 5-Fu application cannot effectively inhibit bleb scarring in AGV implantation. Additionally, our findings regarding the postoperative IOP, central anterior chamber depth, and bleb formation indicate that aqueous humor drainage was kept unobstructed with implantation of the 5-Fu-PCL sustained-release film.

We sutured the drainage tube with one needle using an 8–0 absorbable suture to limit the drainage of the aqueous humor and to prevent the occurrence of continuously low IOP and a shallow anterior chamber, as reported in the literature [20]. Based on the histopathological finding showing that bleb fibrosis of the baseline, roof, and optic nerve sides were minor in eyes treated with 5-Fu-PCL sustained-release film [5], we fixed the 5-Fu-PCL sustained-release film at the rear of the Ahmed drainage disc. The inhibition of proliferation was relatively stronger at this location than that at the limbus side of blebs.

We found that the fibrous tissue surrounding the blebs was thicker than that reported by Nurettin et al. [3]. We hypothesize that this inconsistency may be caused by different antimetabolites. In our study, we chose 5-Fu as the sustained-release drug, while mitomycin was used by Nurettin et al. The capacity of inhibiting cell proliferation is somewhat different between 5-Fu and mitomycin. Mitomycin nearly completely inhibits cell proliferation, whereas 5-Fu only partially inhibits cell proliferation. Moreover, mitomycin has a stronger cell-killing capacity [21] and is more prone to induce conjunctival thinning and conjunctival necrosis in the filtration area than 5-Fu [22].

Early tissue repair after AGV implantation is an important factor affecting the success of the surgery. Bleb scarring after AGV implantation has been shown to be ongoing, and the one-year success rate after AGV implantation declines due to bleb scarring [3]. In the present study, the 5-Fu-PCL sustained-release film was shown to release 5-FU persistently, and the sufficient drug loading capability can guarantee the safety of the experimental animals. These results suggest that the 5-Fu-PCL sustained-release film has the potential to prevent failure of AGV implantation due to bleb scarring.

One limitation of this study is the choice of experimental animal. We chose healthy New Zealand white rabbits as the experimental subjects. These animals had normal aqueous humor drainage pathways compared to the subject with glaucoma. Therefore, aside from drainage by the AGV, a portion of the aqueous humor could also be discharged through the normal channels for aqueous humor outflow after the operation. This condition differs slightly from the real glaucoma condition, in which the aqueous humor drainage pathways are defective to some extent. This fact can be inferred from the observation of the lower postoperative IOP of rabbits in the three groups compared to the preoperative values. Another limitation of this study is that we only observed postoperative bleb scarring for up to 3 months. The presence of persistent scarring after AGV implantation may require a longer term to observe the histopathological changes of the blebs.

## Conclusions

In summary, the 5-Fu-PCL sustained-release film used in the present study fulfills the requirements of sustained-release pharmaceutical preparations. In a rabbit eye model, our results demonstrate that implantation of the AGV with 5-Fu-PCL sustained-release film effectively inhibited bleb scarring.
